# Cross-cultural comparison of breast cancer patients’ Quality of Life in the Netherlands and Japan

**DOI:** 10.1007/s10549-017-4417-z

**Published:** 2017-07-31

**Authors:** M. J. Fischer, K. Inoue, A. Matsuda, J. R. Kroep, S. Nagai, K. Tozuka, M. Momiyama, N. I. Weijl, D. Langemeijer-Bosman, S. R. S. Ramai, J. W. R. Nortier, H. Putter, K. Yamaoka, K. Kubota, K. Kobayashi, A. A. Kaptein

**Affiliations:** 1 Department of Medical Oncology, Leiden University Medical Center, 2300 RC Leiden, P.O. Box 9600, Leiden, The Netherlands; 2 Division of Breast Oncology, Saitama Cancer Center, Saitama, Japan; 3 Department of Hygiene and Public Health, Teikyo University School of Medicine, Tokyo, Japan; 4 Department of Medical Oncology, Medical Center Haaglanden, The Hague, The Netherlands; 5 Department of Pulmonology, Leiden University Medical Center, Leiden, The Netherlands; 6 Department of Medical Statistics, Leiden University Medical Center, Leiden, The Netherlands; 7 Graduate School of Public Health, Teikyo University, Saitama, Japan; 8 Department of Pulmonary Medicine and Oncology, Nippon Medical School, Saitama, Japan; 9 Department of Respiratory Medicine, Saitama International Medical Center, Saitama, Japan; 10 Unit of Psychology, Leiden University Medical Center, Leiden, The Netherlands

**Keywords:** Brief Illness Perception Questionnaire, Breast cancer, Chemotherapy, Cross-cultural comparison, EORTC QLQ-C30, Illness perceptions

## Abstract

**Purpose:**

Cultural differences are hypothesized to influence patients’ Quality of Life (QoL) reports. However, there is a lack of empirical cross-cultural studies comparing QoL of patients with cancer. This study aims to compare QoL of women with breast cancer in the Netherlands and Japan, and to investigate the association of QoL with sociodemographic, clinical, and psychological variables (illness perceptions).

**Methods:**

Dutch (*n* = 116) and Japanese (*n* = 148) women with early breast cancer undergoing chemotherapy completed the EORTC QLQ-C30 and Brief Illness Perception Questionnaire immediately before their second cycle of chemotherapy.

**Results:**

Dutch women reported poorer Physical, Role, Emotional, and Cognitive functioning than Japanese women. Additionally, illness perceptions were significantly different in Japan and the Netherlands, but these did not vary across treatment type. In Japan, QoL of women receiving AC-chemotherapy was better than that of women receiving FEC-chemotherapy, whereas in the Netherlands, QoL did not vary as a function of chemotherapy. Illness perceptions about symptom severity, adverse consequences, and emotional representations were negatively related to most domains of patients’ QoL in both countries. Adding illness perceptions as covariates to the ANOVA analyses rendered the effects of country and treatment type on QoL non-significant.

**Conclusions:**

Comparing Dutch and Japanese women with early breast cancer revealed important differences in treatment modalities and illness perceptions which both appear to influence QoL. Perceptions about cancer have been found to vary across cultures, and our study suggests that these perceptions should be considered when performing cross-cultural studies focusing on patient-reported outcomes.

## Background and rationale

Breast cancer is common in both the East and the West. For adult women in Japan [1] and the Netherlands [2], it is the most common type of malignancy. As a result of improved detection and treatment options, an increasing number of patients survive their breast cancers. As a consequence, patients’ Quality of Life (QoL) has become one of the main outcomes of treatment. Although Japanese and European cancer patients previously have shown considerable equivalence with regard to the concept of QoL [3], cross-cultural comparisons between Asian and European breast cancer patient samples have seldom been performed [4]. One study showed that Japanese women with breast cancer report better physical QoL than German women, but found no difference between countries with regard to emotional well-being [5]. Other studies, with modest sample sizes, revealed no substantial differences in functional domains of QoL between Japanese and Caucasian patients [6, 7].

In order to understand differences in cross-cultural QoL results, the impact of other important predictors of QoL such as sociodemographic characteristics, disease- and treatment-related variables, and psychological factors needs to be considered [8, 9].

Cognitions and perceptions about health and illness vary between cultures [10], which may account for differences in patient outcomes. One model that emphasizes patients’ perceptions in explaining patient reported outcomes is the Common Sense Model (CSM) of Self-Regulation [11]. According to this model, patients’ personal beliefs about the illness and their emotional response determine how individuals will respond to their illness, which in turn affects health outcomes such as QoL. Within the CSM, several dimensions of illness representations are distinguished, such as the symptoms attributed to the illness (Illness Identity), the expected illness duration (Timeline), the consequences for one’s life (Consequences), and the degree to which the illness can be cured or controlled, either by means of medical treatment (Treatment Control) or by the individual himself (Personal Control). It is hypothesized that the emotional response to the illness parallels the illness-related beliefs and cognitions. Recently, Richardson and colleagues performed a meta-analysis about the relationship between cancer patients’ illness perceptions and health outcomes [12]. Consistent inverse associations were found between Physical, Role, Emotional, and Global QoL and several illness perceptions, especially with perceptions about symptom severity, illness consequences, and emotional representations. For patients with breast cancer in particular, several studies have underscored the relationship between illness perceptions and health outcomes [7, 13]. Stronger perceptions about symptom severity and illness consequences have been found to relate to poorer physical functioning [14, 15], whereas less confidence in treatment effectiveness, longer expected timeline, greater symptom severity, negative illness consequences, and intense emotional representations of breast cancer have been related to worse overall emotional well-being [14–20].

Given the strong relation between cancer patients’ illness perceptions and their QoL, examining differences in illness perceptions between cultures is relevant for understanding differences in QoL in cross-cultural studies. To date, few studies have investigated illness perceptions of breast cancer patients with different cultural backgrounds. An exploratory study among Japanese and Dutch women with breast cancer showed that Japanese women reported more concerns about their illness than Dutch women, whereas all other illness perception dimensions were comparable [7]. Cultural backgrounds were also found to affect perceptions about breast cancer in American women [21].

As part of a large randomized trial, investigating the effects of routine monitoring QoL of Dutch and Japanese women with breast cancer receiving chemotherapy, the purpose of the present study was to examine cross-cultural differences in HRQoL between Japanese and Dutch women with early breast cancer, and to investigate the relationship of HRQoL with sociodemographic and clinical variables, and patients’ illness perceptions. As chemotherapy regimens have their typical side effects, we will focus our analyses on differences in functional and general QoL domains, rather than on specific symptoms.

## Methods

### Sample and protocol

This study was performed in accordance with the Helsinki Declaration of the World Medical Association. The study protocol was approved by the institutional review boards of each participating institution. From October 2012 to April 2016, patients were invited by their oncologist before the start of chemotherapy. Inclusion criteria were: female patients with breast cancer stage I–III, performance status 0–1, and scheduled to receive (neo-)adjuvant first-line intravenous chemotherapy. Patients who chose to participate gave written informed consent. Patients filled out an anonymous questionnaire immediately before their second cycle of chemotherapy. Patients completed the questionnaire at the outpatient clinic or at home, and returned it to the clinical research coordinator.

### Chemotherapy

Women in both countries had received a first cycle of one of the following chemotherapy regimens: TAC (75 mg/m^2^ docetaxel, 50 mg/m^2^ adriamycin, 500 mg/m^2^, cyclophosphamide), AC (60 mg/m^2^ doxorubicin, 600 mg/m^2^ cyclophosphamide), FEC (500 mg/m^2^ fluorouracil, 100 mg/m^2^ epirubicin, 500 mg/m^2^ cyclophosphamide), or TC (75 mg/m^2^ docetaxel, 600 mg/m^2^ cyclophosphamide). The combination of concurrent TAC was used in the Netherlands only. In Japan, treatment with AC and taxanes was given sequentially (AC>T).

### Questionnaire

The questionnaire assessed patients’ HRQoL and their illness perceptions. Health-Related Quality of Life was assessed with the European Organization for Research and Treatment of Cancer (EORTC) QLQ-C30 [22, 23]. The 30-item questionnaire consists of five functional scales (Physical, Role, Cognitive, Emotional, and Social) and a Global QoL scale. In addition, the questionnaire comprises nine symptom scales (e.g., fatigue, pain). All the scores for the domains of QoL are transformed into a 0–100 scale. Higher scores on the functional scales and lower scores on the symptoms scales indicate better QoL.

Illness perceptions about breast cancer were assessed with the Brief Illness Perception Questionnaire (BIPQ) [24]. The BIPQ is a validated instrument to assess illness perceptions in various patient groups, including patients with cancer [25]. The BIPQ consists of eight questions that measure eight dimensions of illness perceptions in the following order: Identity (how much do you experience symptoms from your illness), Consequences (how much does your illness affect your life), Timeline (how long do you think your illness will last), Treatment Control (how much do you think your treatment can help your illness), Personal Control (how much control do you feel you have over your illness), Concern (how concerned are you about your illness), Coherence (how well do you feel you understand your illness), and Emotional Representation (how much does your illness affect you emotionally). For this study, the word “illness” was replaced with “breast cancer.” Answers are given on a scale ranging from 0 (not at all) to 10 (very much). The BIPQ can be downloaded from: www.uib.no/ipq.

### Sociodemographic and clinical characteristics

Sociodemographic and clinical characteristics were obtained from patients’ medical records and included age, marital status and employment status, height, weight, body mass index, affected breast(s), cancer subtype, cancer stage, hormone and HER2-receptor status, type and timing (adjuvant or neo-adjuvant) of chemotherapy, and prior radiotherapy.

### Statistical analyses

Descriptive analyses were performed to summarize patients’ sociodemographic and clinical characteristics, QoL domains, and illness perceptions. Possible differences in background variables, QoL, and illness perceptions between Japanese and Dutch patients were examined by means of Chi-square tests or *t* tests. Associations between background variables and illness perceptions with QoL were analyzed by means of Pearson correlations for linear associations, and multivariate analyses of variance (MANOVA) for group mean comparisons. For Japan and the Netherlands separately, MANOVAs were performed to investigate the relation of type of chemotherapy with patients’ QoL. Additionally, for patients receiving the same type of chemotherapy, *t* tests were performed to investigate possible differences in QoL between Japanese and Dutch women.

Similarly for each country, MANOVA was also used to examine a possible relationship between type of chemotherapy and illness perceptions. For patients receiving the same type of chemotherapy, *t* tests were used to investigate possible differences in illness perceptions between Japanese and Dutch women.

Some differences were observed in the types of chemotherapy prescribed in Japan and the Netherlands (see in Results section). Because of this confounding, the variable “Type of chemotherapy” could not be used as a separate control variable in a multivariate analysis. Therefore, it was decided to perform two sets of analyses of variance to investigate whether patients categorized by country and chemotherapy type (six groups) had different functional QoL scores. The first set included only the six groups as a factor in the model (ANOVA). In the second set of analyses, relevant covariates were added into the model (ANCOVA). All analyses were performed using SPSS^®^ 20.0.

## Results

### Patients

**Fig. 1:**
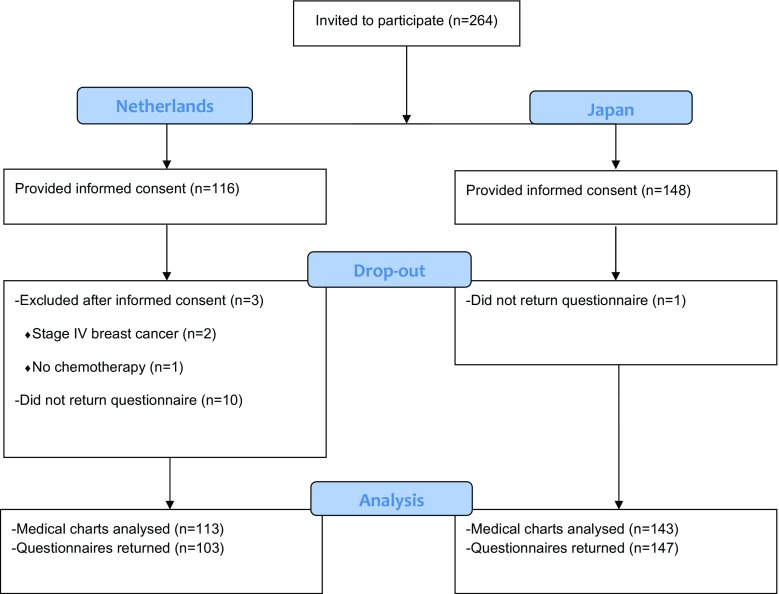
Flow diagram

A total of 264 women (116 Dutch and 148 Japanese) agreed to participate. After informed consent, three patients in the Netherlands were excluded because they did not receive chemotherapy (*n* = 1) or were found to have distant metastases (*n* = 2). Sociodemographic and clinical variables were gathered for 256 patients, and 250 patients returned their questionnaires (Fig. 1).

Sociodemographic and clinical characteristics are shown in Table 1. Whereas breast cancer characteristics were quite similar, treatment details varied to a certain extent between the Netherlands and Japan. AC (doxorubicin + cyclophosphamide)-based chemotherapy was the main choice of chemotherapy in both countries, though in the Netherlands more than half of the patients undergoing AC-chemotherapy received concurrent taxane (TAC-regimen). Also, in the Netherlands more patients received neo-adjuvant chemotherapy and more patients had undergone previous radiotherapy.

**Table 1:** Sociodemographic and clinical characteristics

	Netherlands	Japan	*N*	*p* value
Age (year)^a^	51.9 (10.2)	52.8 (10.2)	256	n.s.
Height (cm)^a^	168.3 (7.2)	156.2 (6.0)	249	<0.001
Weight (kg)^a^	75.2 (16.9)	56.1 (8.9)	250	<0.001
Body mass index (kg/m^2^)^a^	26.5 (5.6)	23.0 (3.5)	249	<0.001
Partnered/married^b^	90 (79.6%)	136 (93.8%)	258	0.001
Employed at time of diagnosis^b^	76 (74.5%)	87 (59.6%)	248	0.015
Affected breast^b^				
Left	47 (41.6%)	62 (43.4%)	256	n.s.
Right	56 (49.6%)	71 (49.7%)		
Bilateral	10 (8.8%)	10 (7.0%)		
Cancer subtype^b^				
Invasive ductal	95 (84.8%)	126 (89.4%)	253	n.s.
Invasive lobular	11 (9.8%)	6 (4.3%)		
Other	6 (5.4%)	9 (6.4%)		
Cancer stage^b^				
I	22 (20.0%)	28 (19.6%)	253	n.s.
II	74 (67.3%)	95 (66.5%)		
III	14 (12.7%)	20 (14.0%)		
ER and/or PR positive^b^	83 (73.5%)	98 (68.5%)	256	n.s.
HER2 positive^b^	23 (20.4%)	41 (28.7%)	256	n.s.
Triple-negative breast cancer^b^	20 (17.7%)	24 (16.8%)	256	n.s.
Timing of chemotherapy^b^				
Adjuvant	55 (48.7%)	105 (73.4%)	256	<0.001
Neo-adjuvant	58 (51.3%)	38 (26.6%)		
1st cycle of chemotherapy^b^				
TAC	48 (42.5%)	0 (0%)	255	<0.001
AC	41 (36.3%)	91 (64.1%)		
FEC	18 (15.9%)	35 (24.6%)		
TC	4 (3.5%)	16 (11.3%)		
PTCptz	2 (1.8%)	0 (0%)		
Previous radiotherapy treatment^b^	30 (26.5%)	16 (10.8%)	261	0.001

^a^Means (SD). Differences in means tested with *t* tests

^b^Frequencies (%). Differences in frequencies tested with *χ* ^2^ tests

### Quality of Life of Dutch and Japanese patients

Overall, internal consistency of QoL scales was somewhat lower in Japan than in the Netherlands (Table 2). The subscales Cognitive Functioning and the Nausea symptom scale in Japan showed Cronbach’s alpha values ≤0.40, so results from these scales should be interpreted with caution. Japanese patients reported higher scores on several of the functional QoL scales compared with Dutch patients. Differences were mostly apparent for Role, Emotional, and Cognitive Functioning. Furthermore, specific symptoms such as fatigue, nausea, and loss of appetite were mentioned more frequently by Dutch women, compared with Japanese women.

**Table 2:** Quality of Life scores in the Netherlands and Japan

	Netherlands		Japan		*N*	*t* test; *p* value
	Cronbach’s α	M (SD)	Cronbach’s α	M (SD)		
EORTC QLQ-C30 function scales						
Physical functioning	0.75	85.8 (13.6)	0.62	89.6 (10.2)	250	0.018
Role functioning	0.86	67.6 (28.9)	0.75	83.3 (19.4)	250	<0.001
Emotional functioning	0.76	76.9 (17.9)	0.66	83.8 (13.6)	250	0.001
Cognitive functioning	0.71	77.7 (22.5)	0.40	89.2 (14.3)	246	<0.001
Social functioning	0.79	75.9 (23.9)	0.64	79.7 (21.0)	250	n.s.
Global quality of life	0.89	69.1 (19.6)	0.86	69.3 (18.9)	250	n.s.
EORTC QLQ-C30 symptom scales						
Fatigue	0.86	40.3 (24.0)	0.75	26.9 (17.5)	250	<0.001
Nausea	0.79	15.5 (25.1)	0.30	4.9 (9.4)	250	<0.001
Pain	0.71	16.3 (22.5)	0.71	13.8 (15.0)	250	n.s.
Dyspnea	n.a.	13.6 (21.6)	n.a.	13.4 (17.7)	250	n.s.
Insomnia	n.a.	33.3 (31.7)	n.a.	17.7 (22.2)	250	<0.001
Appetite loss	n.a.	22.3 (28.9)	n.a.	10.4 (17.4)	250	<0.001
Constipation	n.a.	24.6 (32.0)	n.a.	21.8 (26.6)	250	n.s.
Diarrhea	n.a.	19.1 (32.5)	n.a.	9.3 (18.6)	250	0.007
Financial problems	n.a.	13.3 (26.5)	n.a.	18.8 (24.7)	250	n.s.

### Illness perceptions of Dutch and Japanese patients

Japanese women had remarkably different perceptions of their illness than Dutch women (Table 3). Compared with Dutch women, Japanese women reported that they experienced less severe symptoms (Illness Identity) and less serious consequences of their illness. Whereas Japanese women were less convinced about treatment effectiveness than Dutch women, they believed to have more personal control over their illness. Furthermore, Japanese patients were more concerned about their illness than Dutch women; however, they indicated to be less emotionally affected by their illness.

**Table 3:** Illness perceptions in the Netherlands and Japan

BIPQ scale	Netherlands	Japan	*N*	*t* test; *p* value
	M (SD)	M (SD)		
Consequences	7.3 (2.1)	5.7 (2.8)	249	<0.001
Timeline	5.7 (3.0)	6.4 (2.2)	243	0.045
Personal Control	4.7 (2.9)	6.1 (2.0)	244	<0.001
Treatment Control	8.7 (1.3)	6.9 (2.0)	245	<0.001
Identity	3.8 (2.3)	2.7 (2.2)	249	<0.001
Concerns	5.9 (2.7)	7.1 (2.5)	249	<0.001
Coherence	6.8 (2.4)	6.6 (1.9)	249	n.s.
Emotional Representations	5.0 (2.5)	4.3 (2.6)	249	0.029

### Sociodemographic and clinical factors, and Quality of Life

No associations were found between any of the QoL function scales and patients’ age, marital status or employment. Correlation analyses showed modest but significant inverse associations between patients’ BMI and Physical, Role, Emotional, and Cognitive Functioning (all *r*<−0.20; Table 4). Results also indicated that cancer stage was inversely related to Physical, and Emotional Functioning. Regarding the other clinical factors, multivariate analyses showed that none of the EORTC QLQ-C30 function scales were related to hormone receptor or HER2 status, timing of chemotherapy, or prior radiotherapy (not shown).

**Table 4:** Zero-order correlates of QoL with patients’ clinical characteristics and illness perceptions (235 < *n *< 249)

	PF	RF	EF	CF	SF	GQ
Age	−0.06	0.09	0.06	0.07	0.11	0.00
BMI	−0.15*	−0.15*	−0.17**	−0.17**	−0.02	0.02
Cancer stage	−0.16*	−0.12	−0.15*	−0.11	−0.06	−0.08
BIPQ scale						
Consequences	−0.27***	−0.38***	−0.38***	−0.25***	−0.29***	−0.35***
Timeline	0.00	−0.06	−0.19*	0.01	−0.06	−0.17**
Personal Control	0.07	0.20**	0.25***	0.10	0.14*	0.30***
Treatment Control	0.08	−0.06	−0.02	−0.07	−0.06	0.20**
Identity	−0.40***	−0.48***	−0.28***	−0.30***	−0.29***	−0.49***
Concerns	−0.07	−0.07	−0.33***	−0.04	−0.12	−0.24***
Coherence	0.00	−0.03	0.09	0.03	0.00	0.13*
Emotional Representations	−0.08	−0.23***	−0.53***	−0.21**	−0.21**	−0.29***

*PF* Physical Functioning, *RF* Role Functioning, *EF* Emotional Functioning, *CF* Cognitive Functioning, *SF* Social Functioning, *GQ* Global QoL, *BIPQ* Brief Illness Perception Questionnaire

* *p* < 0.05; ** *p *< 0.01; *** *p* < 0.001

### Illness perceptions and Quality of Life

All functional domains of QoL were negatively related to patients’ intensity of breast cancer-related symptoms (Illness Identity) and perceptions about illness consequences (Table 4). A consistent inverse relation was also observed between patients’ Emotional Representations and their QoL. Patients’ concerns about their illness were negatively related to QoL, although this association was only statistically significant for the domains of Emotional Functioning and Global QoL. Whereas perceptions of Treatment Control were mostly unrelated to the domains of QoL, perceptions of Personal Control showed a weak-to-moderate positive relation to QoL.

In cases where a significant association was observed between a BIPQ domain and QoL, it was examined by means of Fisher *r*-to-*z* transformations whether the strength of the association was similar for both countries. These follow-up analyses showed that associations between illness perception domains and QoL were of similar strength in nearly all (25 of 27 = 93%) comparisons. Two exceptions pertained to a stronger correlation between BIPQ Emotional Representations and EORTC Emotional Functioning in the Netherlands than in Japan (*r* = −0.65 and *r* = −0.40, respectively), and a stronger correlation between BIPQ Consequences and EORTC Social Functioning in Japan than in the Netherlands (*r* = −0.39 and *r* = −0.11, respectively).

### Type of chemotherapy and patients’ Quality of Life

Chemotherapy regimens were included in analyses if at least 15 patients had received a particular treatment. This was done in order to reduce the number of groups in the analyses and to disregard infrequently used types of chemotherapy treatment. In the Netherlands, QoL function domains of patients receiving TAC-, AC- or FEC-chemotherapy were compared. Multivariate (Pillai’s Trace F (12, 174) = 1.61, *p* = 0.09) and univariate comparisons showed no significant effect of chemotherapy on QoL. Moreover, none of the three chemotherapy regimens consistently produced highest or lowest QoL scores across the six function scales, indicating that in the Netherlands differences in QoL between these three groups could not be attributed to the type of chemotherapy used.

In Japan, QoL of patients with AC-, FEC-, or TC-chemotherapy was compared. Although multivariate results did not show statistical significance (Pillai’s Trace F (12, 266) = 1.76, *p* = 0.06), several univariate differences were found for the dimensions Physical, Role, Emotional, and Social Functioning (Table 5), with patients treated with AC-chemotherapy consistently reporting higher QoL than patients receiving FEC-chemotherapy.

**Table 5:** QoL function scales reported per country and chemotherapy regimen

EORTC C30 function scales	Netherlands	Japan	*t* test; *p* value
	M (SD)	M (SD)	
Physical Functioning			
TAC	84.6 (13.2)	–^a^	–
AC	89.0 (11.2)	91.1 (10.2)	n.s.
FEC	89.2 (8.2)	85.1 (10.1)	n.s.
TC	–^a^	89.3 (8.7)	–
	n.s.^b^	0.014^b^	
Role Functioning			
TAC	64.7 (28.9)	–^a^	–
AC	71.6 (26.4)	85.7 (18.3)	0.006
FEC	76.9 (25.7)	76.2 (22.2)	n.s.
TC	–^a^	85.6 (17.7)	-
	n.s.^b^	0.044^b^	
Emotional Functioning			
TAC	80.2 (16.3)	–^a^	-
AC	74.5 (17.4)	85.6 (12.4)	0.001
FEC	79.2 (17.0)	78.3 (16.3)	n.s.
TC	–^a^	79.0 (15.8)	–
	n.s.^b^	0.023^b^	
Cognitive Functioning			
TAC	76.0 (19.7)	–^a^	–
AC	77.8 (26.6)	90.6 (13.9)	0.012
FEC	82.4 (20.2)	85.7 (15.7)	n.s.
TC	**–** ^a^	84.2 (18.0)	-
	n.s.^b^	n.s.^b^	
Social functioning			
TAC	79.1 (23.6)	–^a^	–
AC	76.5 (24.3)	82.8 (19.6)	n.s.
FEC	76.0 (18.3)	70.0 (22.8)	n.s.
TC	–^a^	83.3 (20.9)	–
	n.s.^b^	0.007^b^	
Global QoL			
TAC	68.6 (22.6)	–^a^	–
AC	71.3 (14.7)	70.9 (18.7)	n.s.
FEC	71.5 (15.8)	66.7 (20.8)	n.s.
TC	–^a^	63.3 (17.5)	–
	n.s.^b^	n.s.^b^	

^a^Only mean scores are displayed if number of observations per group >15

^b^ *p* value for difference in QoL subscales between chemotherapy treatment groups

Differences in QoL were also examined between Dutch and Japanese patients receiving the same type of chemotherapy. *T* tests showed that among patients receiving AC-chemotherapy, Japanese women reported better Role, Emotional, and Cognitive Functioning (Table 5).

### Type of chemotherapy and patients’ illness perceptions

No multivariate or univariate differences were found within both countries regarding patients’ illness perceptions (Table 6), indicating that differences in illness perceptions between these three groups could not be attributed to the type of chemotherapy used.

**Table 6:** Illness perceptions of patients with different chemotherapy regimens

BIPQ scales	Netherlands	Japan	*t* test; *p* value
	M (SD)	M (SD)	
Consequences			
TAC	7.6 (1.7)	–^a^	–
AC	6.9 (2.3)	5.4 (2.8)	0.003
FEC	6.4 (2.1)	6.5 (2.6)	n.s.
TC	–^a^	6.1 (2.9)	–
	n.s.^b^	n.s.^b^	
Timeline			
TAC	5.5 (3.0)	–^a^	–
AC	5.8 (2.7)	6.3 (2.2)	n.s.
FEC	5.4 (3.4)	6.7 (2.4)	n.s.
TC	–^a^	5.8 (2.4)	–
	n.s.^b^	n.s.^b^	
Personal Control			
TAC	5.0 (2.9)	–^a^	–
AC	4.5 (2.7)	6.3 (2.1)	0.001
FEC	4.8 (3.1)	5.7 (2.0)	n.s.
TC	–^a^	5.7 (2.1)	–
	n.s.^b^	n.s.^b^	
Treatment Control			
TAC	8.8 (1.3)	–^a^	–
AC	8.4 (1.3)	6.7 (2.1)	<0.001
FEC	9.1 (1.1)	7.4 (1.8)	<0.001
TC	–^a^	6.7 (1.6)	–
	n.s.^b^	n.s.^b^	
Identity			
TAC	4.0 (2.3)	–^a^	–
AC	3.2 (2.1)	2.7 (2.1)	n.s.
FEC	3.8 (2.2)	3.0 (2.5)	n.s.
TC	–^a^	2.6 (2.2)	–
	n.s.^b^	n.s.^b^	
Concerns			
TAC	5.6 (2.5)	–^a^	–
AC	6.7 (2.4)	7.3 (2.4)	n.s.
FEC	4.9 (3.1)	7.2 (2.6)	0.008
TC	–^a^	6.5 (2.7)	–
	n.s.^b^	n.s.^b^	
Coherence			
TAC	7.0 (2.4)	–^a^	–
AC	6.9 (2.4)	6.5 (2.0)	n.s.
FEC	5.7 (2.7)	6.6 (1.6)	n.s.
TC	–^a^	6.9 (1.7)	–
	n.s.^b^	n.s.^b^	
Emotional Representations			
TAC	4.9 (2.3)	–^a^	–
AC	5.7 (2.4)	4.1 (2.7)	0.003
FEC	4.6 (3.1)	4.6 (2.6)	n.s.
TC	–^a^	4.7 (2.4)	–
	n.s.^b^	n.s.^b^	

^a^Only mean scores are displayed if number of observations per group >15

^b^Manova multivariate F-scores for illness perception domains within each country (between group: chemotherapy) are not significant: *p* = 0.12 the Netherlands and *p* = 0.33 in Japan

However, when comparing Dutch and Japanese patients receiving the same type of chemotherapy, significant differences were found in illness perceptions (Table 6). Differences in illness perceptions for patients with similar chemotherapy correspond to the overall cross-cultural differences shown in Table 3.

### Multivariate model for Quality of Life function domains

Analyses of variance were performed to investigate whether patients, categorized by country and type of chemotherapy, had significantly different functional QoL scores. As TAC-chemotherapy was only used in the Dutch sample and was expected to be the most toxic treatment type, significant overall differences were followed by simple post hoc contrast analyses to investigate whether patients receiving TAC showed poorer QoL than patients in the other groups. In a second set of analyses, these ANOVAs were repeated including relevant covariates (ANCOVA). Univariate analyses had suggested that BMI, cancer stage, and illness perceptions were relevant covariates to include in a multivariate model of QoL.

Results from the ANOVAs showed that the six country-by-treatment groups had different mean scores with respect to Physical, Role, Emotional, and Cognitive Functioning (Table 7). Post hoc contrast analyses confirmed that Dutch women receiving TAC had worse Physical Functioning than Dutch and Japanese women receiving AC-chemotherapy (*p*=0.04 and *p*<0.001, respectively). With regard to Role Functioning, Dutch women receiving TAC-chemotherapy performed worse than Dutch women receiving FEC-chemotherapy (*p* = 0.04) and all three Japanese groups (all *p*<0.02). Emotional Functioning of Dutch women treated with TAC was lower than that of Japanese women receiving AC (*p* = 0.01) but not different from the other groups. Finally, Cognitive Functioning of the Dutch group treated with TAC was worse than that of Japanese women receiving AC-chemotherapy (*p*<0.001) or FEC-chemotherapy (*p*=0.02).

**Table 7:** Analysis of variance (1) and co-variance (2) for six domains of functional QoL

		Country-by-Chemotype group	Cancer stage	BMI	BIPQ Consequences	BIPQ Identity	BIPQ Timeline	BIPQ Personal Control	BIPQ Treatment Control	BIPQ Concerns	BIPQ Coherence	BIPQ Emotional Represent.
		*F* value (*p* value)	*F* value (*p* value)	*F* value (*p* value)	*F* value (*p* value)	*F* value (*p* value)	*F* value (*p* value)	*F* value (*p* value)	*F* value (*p* value)	*F* value (*p* value)	*F* value (*p* value)	*F* value (*p* value)
PF												
Without covariates		3.38 **(0.006)**	–	–	–	–	–	–	–	–	–	–
With covariates		1.92 (0.09)	2.48 (0.12)	2.45 (0.12)	2.61 (0.11)	25.60 **(0.000)**	–	–	–	–	–	–
RF												
Without covariates		6.59 **(0.000)**	–	–	–	–	–	–	–	–	–	–
With covariates		2.10 (0.07)		0.63 (0.43)	7.52 **(0.007)**	33.75 **(0.000)**	–	0.01 (0.94)	–	–	–	0.12 (0.73)
EF												
Without covariates		**3.43 (0.005)**	–	–	–	–	–	–	–	–	–	–
With covariates		1.85 (0.10)	5.63 (0.02)	0.61 (0.44)	0.35 (0.56)	0.33 (0.57)	0.11 (0.74)	1.04 (0.31)	–	6.68 (0.01)	–	25.63 **(0.000)**
CF												
Without covariates		**4.96 (0.000)**	–	–	–	–	–	–	–	–	–	–
With covariates		2.06 (0.07)	–	0.88 (0.35)	0.47 (0.49)	**9.07 (0.003)**	–	–	–	–	–	0.91 (0.34)
SF												
Without covariates		2.09 (0.07)	–	–	–	–	–	–	–	–	–	–
With covariates		1.91 (0.09)	–	–	5.13 (0.02)	7.23 **(0.008)**	–	0.01(0.91)	–	–	–	0.40 (0.53)
GQ												
Without covariates		0.71 (0.62)	–	–	–	–	–	–	–	–	–	–
With covariates		1.50 (0.18)	–	–	7.52 **(0.007)**	40.56 **(0.000)**	0.23 (0.63)	8.43 **(0.004)**	5.58 (0.02)	0.00 (0.97)	0.51 (0.48)	0.28 (0.60)

Bold *p* values are significant after Bonferroni correction for multiple testing *p* < 0.05/6 = 0.0083

*PF* Physical Functioning, *RF* Role Functioning, *EF* Emotional Functioning, *CF* Cognitive Functioning, *SF* Social Functioning, *GQ* Global QoL

Next, the analyses were repeated including the relevant covariates (ANCOVA). Differences in the QoL domains between the six country-by-treatment groups were no longer significant after covariates were added (Table 7). In the ANCOVAs, strong associations were found between illness perceptions and QoL. Symptom severity (Illness Identity) was strongly associated with most domains of QoL, with the exception of Emotional Functioning. Perceiving more negative consequences was associated with poorer Role Functioning and Global QoL. Emotional Representations and Concerns about breast cancer were associated with worse Emotional Functioning. Finally, perceptions about Personal Control with respect to breast cancer were strongly related to Global QoL.

## Discussion

This study adds to the sparse body of research that compares QoL between patients with breast cancer from different cultures. The main finding of this study is that, compared with Dutch women, Japanese participants reported better Physical, Role, Emotional, and Cognitive Functioning, after one cycle of chemotherapy. By investigating possible variables that could explain differences in patients’ HRQoL, the present study adopted a biopsychosocial perspective. These analyses have suggested that treatment regimens and patients’ perceptions about breast cancer are different in both countries, and that these factors are highly relevant in understanding the differences in QoL between Japanese and Dutch women.

Effects of chemotherapy regimens on breast cancer patients’ QoL have been reviewed extensively [26]. In the present study, patients were not randomly allocated to receive a specific type of chemotherapy. Instead, patients’ physical condition, patient preferences, and hospital treatment standards have influenced the choice of chemotherapy. For instance, in the Japanese sample, TAC was not used as oncologists wish to reduce the chance of toxicity-induced treatment drop-out. TAC is considered a more toxic treatment than sequential AC>T therapy, which could partly explain the difference in HRQoL between the Japanese and Dutch women found in this study.

Interestingly, whereas most Japanese women with breast cancer reported higher scores on most of the functional domains of HRQoL than Dutch women, Global QoL scores were similar between both countries, for which several explanations can be offered. Firstly, when considering one’s Global QoL, it is likely that the individual takes into account more aspects than perceived health. These unknown variables may include financial income, housing conditions, or social support. It is possible that these variables are different for the Netherlands and Japan. In addition, general subjective well-being may be determined by other predictors in the East than in the West. For instance, in Japan, the quality of close relationships may contribute more to well-being than in European societies. By contrast, individual achievements in terms of financial success or career may be more important in western societies than in Japan [27]. One final explanation concerns the difference in wording between the Global QoL scale (item 29 and 30) and the other items of the EORTC QLQ-C30. Whereas for items 1–28, the questions are phrased in a negative direction (patients’ problems), questions 29 and 30 are phrased in a positive direction (patients’ satisfaction). It is known that people in Eastern Asia are more used to using both negative and positive descriptions when referring to themselves (“dialectical thinking” [28]) and exhibit more ambivalent responding on self-report items, whereas western individuals will show more orthogonal response styles when answering positively and negatively framed questions [29]. This would imply that symptom severity and functional problems can be expected to show a weaker association with overall life satisfaction (Global QoL) in Japan than in the Netherlands. Additional analyses performed on the present study sample found preliminary support for this hypothesis. If this were the case, this would raise questions about the cross-cultural validity, especially the metric equivalence, of the EORTC QLQ-C30 [23, 30].

In support of the CSM [11] and previous studies in the field of oncology [12], this study showed that illness perceptions were strongly associated with several domains of QoL. Additionally, this study found that Japanese women with breast cancer held very different illness perceptions in comparison with Dutch women, although both samples were similar with regard to breast cancer characteristics. These findings are in line with previous studies that investigated illness perceptions in Japanese and Dutch patients with breast cancer [7, 31]. Dutch patients reported experiencing more severe symptoms and more negative consequences than Japanese women. These two illness perception domains were strongly related to most functional domains of QoL in our multivariate analyses, confirming recent findings by Richardson and colleagues [12]. This study also showed that perceptions about the effectiveness of medical treatment for breast cancer were less optimistic in Japan than in the West, as has been found previously [32]. By contrast, Japanese patients reported higher means for personal controllability than Dutch women. Whereas both types of perceived control have been found to be associated with better QoL [12], correlation analyses in the present study showed that perceptions of Personal Control were more strongly related to QoL than perceptions about Treatment Control.

We explored how illness perceptions varied as a function of the type of chemotherapy. Interestingly, in both countries, neither perceived symptom severity (Illness Identity) nor any other of the BIPQ domains varied as a function of chemotherapy type. This suggests that inter-individual variations within each treatment group are more predictive of QoL than differences between treatment groups.

## Limitations

In the present study, analyses were cross-sectional, which prevents making statements about causality. Longitudinal analyses that assess illness perceptions before QoL may provide more support for the hypothesized effect of illness perceptions on patient-reported outcomes. Secondly, we did not assess QoL before the start of treatment. As this study was part of a larger investigation about the effects of monitoring QoL in breast cancer patients during chemotherapy [33], all patients had received their first cycle of chemotherapy before filling out the questionnaire. Selecting patients before chemotherapy and including a baseline assessment of QoL before chemotherapy would have helped to identify to what extent different chemotherapy modalities affect QoL. Finally, our study showed that although nearly all participants (233/255 = 91%) had received anthracycline + cyclophosphamide-based (‘third generation’) chemotherapy, concurrent AC with taxane treatment (TAC-regimen) was not prescribed in the Japanese sample, whereas it was often used in the Netherlands. Therefore, we could not investigate how this TAC-regimen affected QoL in both countries. Although some studies have suggested that the addition of taxane treatment to an AC-regimen may have a somewhat greater negative impact on global QoL during treatment than AC-regimen without taxanes [34], the present study found no significant effects of chemotherapy type on QoL when covariates such as patients’ individual illness perceptions were controlled for.

## Implications

A relevant objective for future research is to examine why Japanese and Dutch breast cancer patients hold such different perceptions about breast cancer. Following the quantitative results obtained with the BIPQ, qualitative studies are needed to reveal what patients think of when they consider, for example, their symptoms, consequences, and controllability of breast cancer. This will answer the question whether the observed differences in illness perceptions are merely a matter of quantity or reflect underlying conceptual differences between Japanese and Dutch patients.

Regarding practice implications, results from this study suggest that promoting patients’ sense of control over the illness (e.g., symptoms and treatment side effects) may improve patients’ emotional well-being and global perceptions about QoL [35]. Educational interventions about treatment and side effects, stress-reduction interventions, such as mindfulness and relaxation, and peer support programs may contribute to patients’ sense of mastery, which in turn may increase their emotional well-being and overall sense of QoL. Additionally, discussion and adequate treatment of physical symptoms and treatment side effects should be a major clinical goal during any type of chemotherapy, as patients’ perceptions about symptom severity are entwined with nearly all domains of HRQoL.

## Conclusions

The results of this study show that there are important differences in HRQoL between Dutch and Japanese women with breast cancer receiving chemotherapy. Our results suggest that these differences may partly be explained by differences in treatment regimens, but even more by the differences in how Japanese and Dutch patients perceive their illness. Perceptions about cancer vary between cultures [10] and our study suggests that these perceptions should be considered when performing cross-cultural studies focusing on patient-reported outcomes.
